# TLR2 Controls Intestinal Carcinogen Detoxication by CYP1A1

**DOI:** 10.1371/journal.pone.0032309

**Published:** 2012-03-19

**Authors:** Khoa Nguyen Do, Lisbeth Nielsen Fink, Thomas Elbenhardt Jensen, Laurent Gautier, Alexandr Parlesak

**Affiliations:** 1 Center for Biological Sequence Analysis, Technical University of Denmark (DTU), Lyngby, Denmark; 2 DTU Multiassay Core (DMAC), Technical University of Denmark (DTU), Lyngby, Denmark; 3 Department of Exercise and Sport Sciences, University of Copenhagen, Copenhagen, Denmark; 4 Metropolitan University College, Global Nutrition and Health, Copenhagen, Denmark; Enzo Life Sciences, Inc., United States of America

## Abstract

Intestinal cytochrome P450 subclass 1A1 (CYP1A1) contributes to a metabolic “shield” protecting the host from ingested carcinogens such as polycyclic aromatic hydrocarbons (PAH). The expression of CYP1 (including CYP1A2 and CYP1B1) is considered to depend solely on a heterodimeric transcription factor consisting of the arylhydrocarbon receptor (AHR) and the AHR nuclear translocator (ARNT). So far, no interference has been noted between the regulation of CYP1 and the activation of Toll-like receptor 2 (TLR2), which modulates the inflammatory response to bacterial cell wall components in immune cells and enterocytes. Here we report that intestinal CYP1A1 is silenced in TLR2-deficient mice, even when under exposure to the carcinogenic PAH benzo[a]pyrene (BaP). In contrast, hepatic CYP1A1 was moderately induced in TLR2-deficient mice without restoring their ability to clear BaP from systemic circulation, as present in wild-type animals. After feeding of BaP for 21 days, only TLR2^−/−^ mice, but not their wild type littermates developed polyps in the colon. Gene expressions and protein concentrations of AHR and ARNT in the intestine did not differ between the genotypes. In conclusion, the presence of ligands for TLR2 of bacterial origin seems to be crucial for detoxication of luminal carcinogens by CYP1A1 in the intestine. This unprecedented finding indicates a complex interplay between the immune system of the host and intestinal bacteria with detoxication mechanisms. This highlights the relevance of intestinal microbiota when trying to unravel pathways present in mammals and opens new perspectives for research in human health.

## Introduction

The toll-like receptor (TLR) family comprises a group of type 1 transmembrane glycoproteins, which recognize microbe-associated molecular patterns (MAMPs) [1]. Binding of MAMPs to these receptors induces innate immune responses such as cytokine production, phagocytosis initiation, antigen presentation and other molecular events resulting in inflammation and its autoregulation [2]. Toll-like receptor 2 (TLR2) in association with TLR1 or TLR6 is crucial for recognition of bacterial lipoproteins, lipopeptides, and other microbial cell wall components [3] and consequent induction of pro-inflammatory responses predominantly in polymorphonuclear leukocytes and phagocytes [4]. TLR2, together with TLR1 or TLR6, signals as part of a heterodimer in association with lymphocyte antigen 96 (MD2) by recruitment of myeloid differentiation primary response protein 88 (MyD88), toll-interleukin 1 receptor (TIR) domain containing adaptor protein (TIRAP), and toll-like receptor adaptor molecule 2 (TICAM2), to their intracellular signaling domains [5]. The TLR2-containing agglomerate binds the intracellular adaptor complex (IRAK1) to trigger the intracellular activation of MAPKs and of the canonical pathways linked to NF-kappaB and phosphoinositide 3-kinase (PI3K)/AKT. The coordinated signaling directs production of immunoregulatory mediators (e.g., IL-1 beta, TNF-alpha) [6]. Some bacteria, which are considered to have probiotic properties and harbour ligands for TLR2, have been discussed as protective agents in colon cancer development [7].

Polycyclic aromatic hydrocarbons (PAH) such as benzo[a]pyrene (BaP) and phenanthrene are cancer-inducing agents found in tobacco smoke, automobile emission exhaust, industrial waste, and even certain foodstuffs [8]. Data from epidemiological studies, biochemical and molecular investigations support the idea of PAH such as BaP to be primary causative carcinogens in the lung [9] and the bladder [10]. The key reactive intermediate benzo(a)pyrene-7,8-diol-9,10-epoxide (BPDE) can link covalently to DNA, therefore causing genetic aberrations [10]. BPDE is formed endogenously in a multi-step procedure in which cytochrome P450 (CYP) subclasses CYP1A1 and CYP1B1 are of special relevance in both metabolic activation (epoxidation) and detoxication [9]. The regulation of CYP1 is considered so far to be unrelated to TLR2 activation [11]. The expression of CYP1 is under control of the cytosolic arylhydrocarbon receptor (AHR) [12], which heterodimerizes upon ligand (e.g., PAH, dioxin) binding with the aryl hydrocarbon receptor nuclear translocator (ARNT). This heterodimer forms a high-affinity DNA binding complex that binds to specific target DNA sequences known as aryl hydrocarbon receptor-response elements (AHRE) in the regulatory areas of the responsive CYP1 genes [13].

In an initial experiment, we identified the basal expression of CYP1A1 to be dramatically reduced in the intestine of TLR2^−/−^ mice compared to wild-type (WT) animals with the corresponding genetic background. In a subsequent feeding study, we proved that feeding with BaP does neither induce intestinal CYP1A1 mRNA nor protein expression in TLR2^−/−^ mice in contrast to WT mice and that TLR2^−/−^ animals are incapable of systemic BaP clearance.

## Materials and Methods

### Animals

C57BL/6 WT mice and B6.129-TLR2^tm1Kir^/J mice were obtained from the Jackson Laboratory, Bar Harbor, Maine, USA. B6.129-TLR2^tm1Kir^/J mice are homozygous for the TLR2^tm1Kir^ mutation and do not produce functional TLR2 protein [14]. Mice were 13–14 weeks at the start of the experiment. Animals were kept under 12 h-light-dark cycles at 22°C.

Mice were handled by experienced animal keepers and all protocols and procedures applied were approved by the representative of the Danish National Committee on Animal Experimentation (Forsøgsdyrtilsynet). Special care was taken to minimize any suffering of the mice.

### Feeding experiment

Mice had *ad libitum* access to water and chow (Altromin 1324 pellets, Brogaarden, Gentofte, Denmark) that was soaked for 24 hours with corn oil (1 mL corn oil/12 g chow), which either contained benzo[a]pyrene (BaP, Sigma-Aldrich, Brøndby, Denmark) (5.8 mg/g corn oil) or not. In the treatment groups, the daily intake was adjusted to be 100 mg BaP/kg/day [15], based on the average daily chow consumption of mice. Mice of each genotype (wild-type and TLR2^tm1Kir^/J, 30 in total) were divided into two groups (7–8 animals/group), one of them receiving BaP. Mice were euthanized at 21 days after pentobarbital sedation by exsanguination.

### Collection of plasma and tissues

Blood samples were collected in EDTA-treated syringes (Kruuse, Langeskov, Denmark) and centrifuged (3000×g, 10 min, 4°C). The supernatant plasma was kept at −80°C before HPLC analysis. Immediately after exsanguination, the small intestines were removed, rinsed with an ice-cold aqueous solution of 1.15% KCl, 1 mM phenylmethanesulfonylfluoride, and 0.5 mM DTT. The epithelial cell layer was scraped off with the edge of a cover glass used for microscopical purposes and RNA was isolated according to the TRIzol Plus RNA isolation protocol (Invitrogen, Taastrup, Denmark). The aqueous phase was transferred to RNA purification spin columns (RNeasy Mini Kit, Qiagen, Hilden, Germany) and was isolated according to the RNeasy protocol.

The liver was first perfused with TRI Reagent (Ambion, Applied Biosystems, Foster City, CA, USA), followed by perfusion next with RNA*later* (Applied Biosystems, Nærum, DK), cut into small pieces, and shock-frozen with liquid nitrogen. The isolated RNA (ABI Prism 6100 Nucleic Acid Prep Station using Nucleic Acid Purification Lysis Solution and Total RNA Purification Trays, Applied Biosystems) was further purified with Total RNA Isolation kit (Agilent).

Concentrations of RNA were measured with NanoDrop ND-1000 spectrophotometer (Thermo Fisher Scientific Inc., Roskilde, DK) or the Quant-iT RNA Assay Kit (Invitrogen) and purity was certified with the BioAnalyzer 2100 (Agilent).

For protein extraction, untreated mucosal and hepatic tissue was shock-frozen with liquid nitrogen. All samples were kept frozen at −80°C until use. Large intestines for histological investigation were flushed free of fecal contents with ice-cold phosphate buffered saline (PBS), cut open along the longitudinal median axis, and fixed between filter paper in 10% formalin.

### Microarray measurements

Two hundred nanograms of total RNA from six mice/group (WT and TLR2^−/−^) were separately converted into labeled cRNA with nucleotides coupled to a Cyanine 3-CTP (Cy3) fluorescent dye using the One-Color Microarray-Based Gene Expression Analysis, Low Input Quick Amp Labeling (version 6.0 protocol) (Agilent Technologies). Cy3-labeled cRNA (1.65 µg) from each mouse was hybridized to Agilent Whole Mouse Genome Oligo Microarrays (six per group) (G4122F) for 17 h at 65°C. The hybridized microarrays were scanned using an Agilent DNA Microarray Scanner with Surescan High-Resolution Technology (G2505C) and evaluated (Agilent Technologies Feature Extraction software version 10.5.1.1).

### qRT-PCR

For cDNA synthesis, total RNA was reverse-transcribed into cDNA using the High-Capacity cDNA Reverse Transcriptase Kit (Applied Biosystems). The reverse-transcription conditions were as follows: 25°C for 10 min, 37°C for 120 min and 85°C for 5 sec in a VWR Unocycler (VWR, West Sussex, UK).

Gene expression assays for qRT-PCR were purchased from Applied Biosystems. Assay IDs were as follows: *Cyp1A1* (Mm00487218_m1), *Cyp1A2* (Mm00487224_m1), *Cyp1B1* (Mm00487229_m1), *Ahr* (Mm01291777_m1), and *Arnt* (Mm00507836_m1). TaqMan Gene Expression Master Mix, (Applied Biosystems) was mixed with 4 µL of diluted first-strand cDNA as template and TaqMan Gene Expression Assay mix (forward primer, reverse primer and FAM™ dye-labeled TaqMan MGB probe) to give a final volume of 10 µL (2.5 µg/µL cDNA) per reaction. The 7900HT Fast Real-Time PCR System (Applied Biosystems) was used for duplicate analyses. Cycle conditions were 2 min at 50°C and 10 min at 95°C, followed by 40 cycles of 15 s at 95°C and 1 min. at 60°C. Assays specific for *β-actin* (Mm01205647_g1) and *Gtf3c2* (Mm00510828_m1) were used as the reference genes to account for variability in the concentration of cDNA (Genevestigator software) [16]. The threshold cycle (ΔΔCt) method of comparing expression data was applied. Gene expression was normalized to Ct values of *β-actin*/*Gtf3c2* [ΔCt = Ct(target)-Ct(reference)] and comparative gene expression was calculated as ΔΔCt = ΔCt(treatment)-ΔCt(control). The fold change values were calculated as 2^−ΔΔCt^.

### Immunoblot analysis

Microsomes from liver and small intestines were prepared from samples homogenized in 3 mL of 1.15% KCl, 1 mM PMSF, and 0.5 mM DTT to 200 mg of mucosal tissue and 500 mg of hepatic tissue by differential ultracentrifugation (70 Ti rotor in a Beckmann Coulter L8-70M centrifuge): 20 min at 16,000×*g* (4°C) pre-centrifugation and 100,000×*g* for 60 min. The resulting pellet was suspended in 1 mL of 10 mM Tris-HCl buffer with 1 mM EDTA and 1.15% KCl, pH 8.0, aliquoted, and stored at −80°C until use. Protein concentrations were determined at 280 nm using NanoDrop ND-1000 spectrophotometer. Equal amounts of microsomal proteins were separated on Bis-Tris polyacrylamide gels (NuPAGE 4–12% Bis-Tris, Invitrogen) under denaturating conditions and transferred on PVDF membranes (Immobilon–P, Millipore, Bedford, MA, USA) in NuPAGE transfer buffer (Invitrogen). Membranes were then blocked with 0.5% bovine serum albumin dissolved in TBS (20 mM Tris-HCl, pH 7.3, 150 mM NaCl) containing 0.1% Tween-20 and 0.02% NaN_3_). The membrane was incubated with polyclonal rabbit anti-mouse CYP1A1/2 (ARP41405, Nordic Biosite, Täby, Sweden) and polyclonal rabbit anti-mouse antibodies. PTGS2 (PAB6665, Abcam, Cambridge, UK) diluted in TBS-T was included as reference protein [17]. After triplicate washes in TBS-T, the membrane was incubated with a horseradish peroxidase-conjugated secondary antibody (goat-anti-rabbit, Abcam). Relative protein concentrations of AHR and ARNT were measured after lysis of mucosal cells (T-PER Tissue Protein Extraction Reagent with Halt Protease Inhibitor Cocktail, Thermo Scientific, Herlev, Denmark) from mice of both genotypes. Primary antibodies used were AHR polyclonal antibody (goat-anti-mouse AHR, PAB12101), ARNT monoclonal antibody, clone H1beta234 (MAB2370) (both: Abnova), and polyclonal rabbit-anti-mouse α-actin antibody (A2668, Sigma-Aldrich). Secondary antibodies were polyclonal (anti-mouse IgG, anti-goat IgG, and anti-rabbit IgG, all peroxidase-conjugated, DAKO, Glostrup, Denmark). Protein bands were visualized using enhanced chemiluminescence reagent (ECL, Amersham, Buckinghamshire, UK). Relative chemoluminescence intensities of CYP1A1, CYP1A2, AHR, and ARNT protein were normalized.

### Histology

The colon of mice under investigation was examined for presence of aberrant crypt foci and other morphological abnormalities. After 24 hours in fixative, colons were stained with 0.2% methylene blue (VWR, Leuven, Belgium) in 10% formalin solution for 30 min and washed with PBS/methanol 80/20 v/v. The tissue was examined with an Olympus BX51 light microscope equipped with an Olympus DP20 digital color camera (40-fold magnification).

### HPLC analysis of benzo[a]pyrene (BaP)

BaP was extracted by a previously established extraction method for lipophilic compounds from blood plasma [18]. In brief, 200 µL of ethanol (p.a., Merck, Darmstadt, Germany) were added to 400 µL of plasma. After homogenization, 500 µL of toluene (p.a., Merck) containing chrysene (Sigma-Aldrich, 25 µg/mL) as internal standard were added. After homogenization and phase separation, the toluene phase was transferred into fresh tubes. The solvent was evaporated and the residue was dissolved in 100 µL of acetonitrile (ACN), 20 µL of which were directly applied to the HPLC system (Waters™ 717plus autosampler, Waters™ HPLC pump 510, Waters degasser, and a Varian 9070 Fluorescence detector with a Luna 5 µm C18(2) column, Phenomenex, Allerød, Denmark); flow was 1.0 mL/min, excitation/emission wavelengths 264/434 nm, resp. The specifity of the BaP analysis procedure was ascertained with a BaP reference standard (TraceCERT®, Sigma-Aldrich). The chromatograms were evaluated using the Millenium 2010 v.3.05 software package. The BaP concentrations were calculated taking the recovery of chrysene into account.

### Statistical analysis

Microarray data obtained from image analysis were processed with the Limma package [19] including background correction and quantiles normalization. A linear model was fitted and modified t-statistics (empirical Bayes shrinkage) was applied using the default proportion and limits for standard deviation (0.01 and [0.1, 4] respectively). The fold-changes reported are ratios of the averages calculated for the wild-type and TLR2^−/−^ animals. *P* values were calculated for the group of genes the averaged expression of which differed by a factor of at least 4.

Significances of differences for inter-group comparisons of PCR, immunoblot data, and circulating BaP concentrations were calculated by two-way ANOVA and Tukey's post-hoc test (Statistica v.9.0 package, StatSoft, Tulsa, OK, USA), partly with logarithmically transformed data.

## Results

In the first experiment, differences in gene expression in the intestines between mice that were deficient for functional TLR2 protein and wild type (WT) animals were measured with Agilent Whole Mouse Genome Oligo Microarrays. As evident from the results of the microarray analysis (Table 1), 14 genes were up- or down-regulated at least 4-fold in the intestines of the TLR2-deficient animals compared to those in WT mice. Out of all measured gene expressions, that of the *Cyp1a1* gene was changed the most – its expression was negligible in the intestines of mice that did not express functional TLR2 (22-fold reduction compared to WT animals). Of note, the expression of mRNA for phospholipase A2 [IVC cytosolic, calcium-independent - *Pla2g4c*, as assayed by probes targeting the sequences NM_001004762 (RefSeq) and AK145339 (Genbank)], was significantly increased in the intestine as a result of the loss of functional TLR2.

**Table 1:** Relative gene expressions in the intestine of TLR2-deficient that are changed at least 4-fold compared to wild-type mice.

Probe set	Target ID	Gene symbol	Gene name	FC	Raw*P* value	Adjusted*P* value
	Up-regulated					
A_52_P17207	AK145339	*Pla2g4c*	phospholipase A2, group IVC (cytosolic, calcium-independent)	9.6	1.23×10^−2^	3.06×10^−1^
A_51_P224263	NM_134052	*Adi1*	acireductone dioxygenase 1	9.3	2.95×10^−10^	1.22×10^−5^
A_51_P428360	NM_023881	*Retnlb*	resistin like beta	9.2	1.97×10^−2^	3.43×10^−1^
A_52_P243075	NM_001004762	*Pla2g4c*	phospholipase A2, group IVC (cytosolic, calcium-independent)	7.7	1.67×10^−2^	3.30×10^−1^
A_52_P228437	AK088871	*Mbnl1*	muscleblind-like 1 (Drosophila)	5.1	5.22×10^−7^	1.13×10^−3^
A_52_P203440	U90926	*U90926*	cDNA sequence U90926	5.0	3.85×10^−3^	2.30×10^−1^
	Down-regulated					
A_51_P279693	NM_009992	*Cyp1a1*	cytochrome P450, family 1, subfamily a, polypeptide 1	−22.5	1.23×10^−4^	5.84×10^−2^
A_52_P731333	AK042046			−14.1	2.12×10^−8^	1.25×10^−4^
A_52_P520788	AK039908	*Scfd1*	sec1 family domain containing 1	−10.3	5.50×10^−9^	5.68×10^−5^
A_51_P215627	NM_207229	*Plac9*	placenta specific 9	−8.8	5.39×10^−9^	5.68×10^−5^
A_51_P162984	NM_013637	*Prm1*	protamine 1	−7.3	1.26×10^−3^	1.62×10^−1^
A_51_P423290	XM_284198	*Mmrn1*	multimerin 1	−7.1	1.24×10^−6^	2.56×10^−3^
A_52_P565847	NM_001004153	*AU018091*	expressed sequence AU018091	−4.9	3.89×10^−2^	4.08×10^−1^
A_51_P194099	NM_009381	*Thrsp*	thyroid hormone responsive SPOT14 homolog (Rattus)	−4.2	2.08×10^−2^	3.46×10^−1^

RNA was isolated from intestines of six wild-type and six TLR2^−/−^ mice and gene expression was measured by applying cRNA from each mouse individually on an Agilent Whole Mouse Genome Oligo Microarray (G4122F). CYP1A1 expression was nearly absent (−96%) in animals deficient for the *Tlr2* gene.

**Figure 1:**
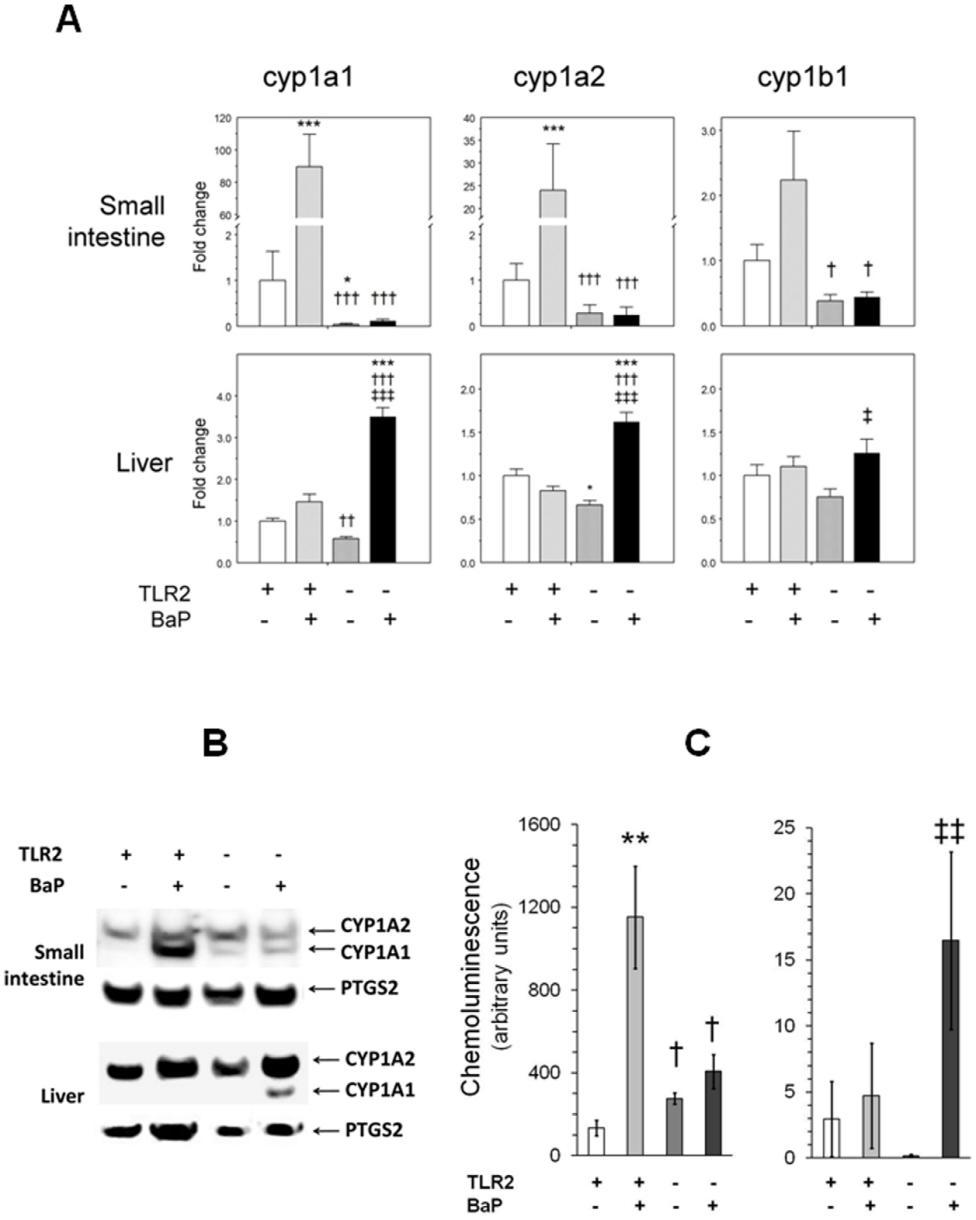
Expression of mRNA and protein production of hepatic and intestinal CYP1A1, CYP1A2, and CYP1B1. (A) Gene expression levels for cytochrome P450 isoforms 1A1, 1A2 and 1B1 in the intestine and liver of mice that were either deficient for TLR2 (TLR2^−/−^) or not and that were either fed with chow soaked with corn-oil with benzo[a]pyrene (BaP) or without (n = 7–8 animals/group). Analyses were performed by qRT-PCR and statistical analysis was performed by two-way ANOVA followed by Tukey's post-hoc test. Values are given as means ± SEM; *(**/***): *P*<0.05 (0.01/0.001, resp.) vs. WT group without BaP feeding; †(††/†††): *P*<0.05 (0.01/0.001, resp.) vs. WT group with BaP feeding; ‡ (‡‡/‡‡‡): *P*<0.05 (0.01/0.001, resp.) vs. TLR2^−/−^ group without BaP feeding; (B) Immunoblot analyses of protein expressions of CYP1A1 and CYP1A2 in the small intestine and the liver of mice that were either deficient for TLR2 (TLR2^−/−^) or not and were either fed with BaP for 3 weeks (100 mg/kg/day) or not. For all proteins investigated, lanes were loaded with 50 µg of microsomal protein per lane. Prostaglandin-endoperoxide synthase 2 (PTGS2) was used as a loading standard. (C) Densitometric evaluation of chemoluminescence of CYP1A bands: BaP feeding induced massively CYP1A1 protein production in the intestine of wild-type animals, but not in that of TLR2^−/−^ mice. In contrast, CYP1A1 was induced moderately in the liver of TLR2^−/−^ animals, but not in that of wild-type mice.

In the consequent intervention study, we investigated whether feeding with BaP induces expression of intestinal mRNAs for CYP1A1, CYP1A2, and CYP1B1 as well as concentrations of CYP 1A1/CYP1A2 protein in TLR2^−/−^ mice and in wild-type animals. After the end of the feeding period (21 days), regulation of mRNA production for CYP1 was measured by qRT-PCR. In the intestines of WT animals, BaP feeding induced the expression of CYP1A1 mRNA to be approx. 90-fold and that of CYP1A2 mRNA to be approx. 24-fold (Figure 1A). In contrast, neither CYP1A1 mRNA nor CYP1A2 mRNA expressions were significantly changed in the intestines of the TLR2^−/−^ animals. The expression of CYP1B1 mRNA did not change significantly in the intestines of BaP-fed WT mice (Fig. 1A). In contrast to the intestine, BaP feeding enhanced the hepatic expression of CYP1A1 mRNA and CYP1A2 mRNA approx. 3.5-fold and 1.5-fold, respectively, in TLR2^−/−^ animals, but not in that of the WT mice (Fig. 1A). The expression of mRNA for the arylhydrocarbon receptor (AHR) and its heterodimeric partner, arylhydrocarbon receptor nuclear translocator (ARNT) differed neither in the intestine nor in the liver of WT or TLR2^−/−^ animals under any feeding regimen (Table 2).

**Table 2:** Hepatic and intestinal mRNA expression of AHR and ARNT in TLR2^−/−^ and wild-type mice with and without BaP feeding.

	AHR				ARNT			
TLR2	+	+	−	−	+	+	−	−
BaP	−	+	−	+	−	+	−	+
Intestine	1.00 (0.43)	1.41 (0.42)	1.67 (1.01)	2.65 (3.17)	1.00 (0.32)	1.05 (0.19)	1.01 (0.43)	1.48 (0.34)
Liver	1.00 (0.16)	0.78 (0.15)	0.64 (0.17)	0.73 (0.18)	1.00 (0.10)	0.72 (0.10)	0.73 (0.08)	1.04 (0.15)

None of the values differs significantly from another (2-way ANOVA, group-wise comparisons for organs and genes). Values are averages (standard deviation) and are standardized towards *Gtfc2* and *β-actin*.

Quantitative RT-PCR confirmed the finding of the microarray analysis that basal mRNA expression of CYP1A in the intestine was significantly lower in TLR2^−/−^ mice (∼1% of control). Without reaching significance, the average protein concentration of CYP1A1 was moderately higher in TLR2^−/−^ animals compared to WT mice that were not exposed to BaP (Fig. 1B/C). In parallel to the mRNA expression, BaP feeding resulted in significantly enhanced expression of CYP1A1 protein in the intestine of WT mice, but not in that of the TLR2^−/−^ animals (Fig. 1B/C). In the liver of the TLR2^−/−^ animals, BaP supplementation enhanced CYP1A1 protein production only in TLR2^−/−^deficient mice (∼100-fold), but not in that of WT animals (Fig. 1B/C). All groups showed a markedly higher concentration of CYP1A2 in the liver compared to the intestine. The expression of this protein was not significantly affected by BaP feeding either in WT or in TLR2^−/−^ animals.

**Figure 2:**
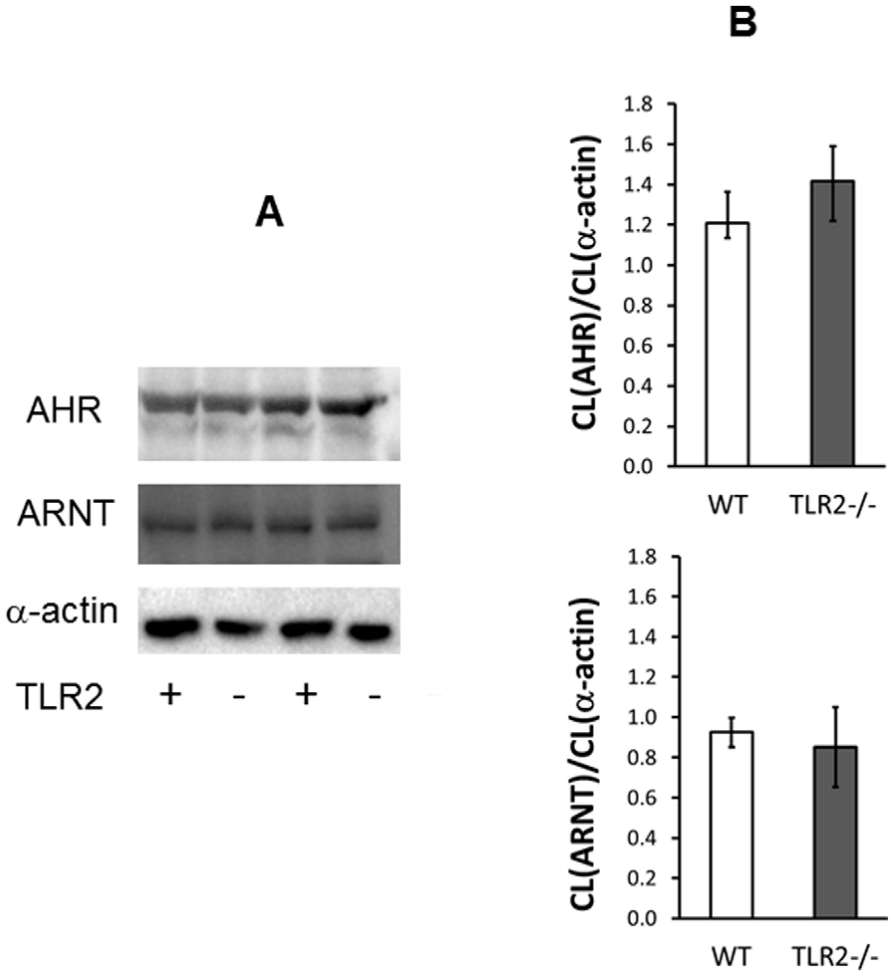
(A) Representative Western blots and (B) standardized protein expression of the aryl hydrocarbon receptor (AHR) and the aryl hydrocarbon receptor nuclear translocator (ARNT). The protein expression in the intestinal mucosa of TLR2^−/−^ mice and in that of WT mice (n = 5 animals/group) was normalized to α-actin. Statistical analysis was performed with Student's t-test. The expression of both proteins did not differ significantly between the genotypes (*P* = 0.394 for AHR and 0.737 for ARNT); CL: chemoluminescence.

Protein expressions of AHR and ARNT did not differ significantly between TLR2^−/−^ and WT mice and molecular masses of both proteins were identical in both genotypes (Figure 2).

**Figure 3:**
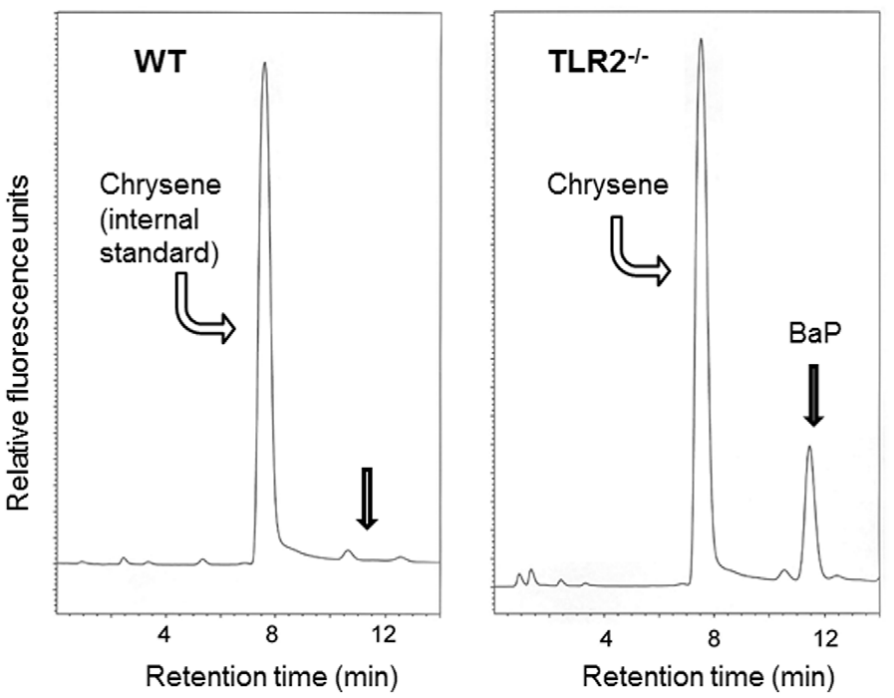
Representative HPLC chromatograms of the blood plasma extract of WT mice (A) and TLR2^−/−^ mice (B). In contrast to WT animals, mice that were deficient for TLR2 were incapable to eliminate BaP from their circulation after an over-night fast. The major peak in both chromatograms was caused by the internal standard chrysene (retention time: 7.5 min). Chromatograms of the blood plasma from TLR2^−/−^ animals (n = 7 per group) showed a clear BaP peak (right chromatogram, retention time 11.5 min) after over-night (16 h) deprivation from BaP-charged chow, while the blood plasma of the WT animals was virtually free of BaP (left chromatogram, concentration below 0.05 ng/mL).

After overnight deprivation of the BaP-loaded chow, the blood plasma of all WT animals was virtually free of BaP (concentration below detection limit, ∼0.05 ng/mL), while the TLR2^−/−^ mice were incapable of clearance of the absorbed BaP (conc. = 61.7±10.5 ng/mL, n = 7, *P*<0.001; Fig. 3).

**Figure 4:**
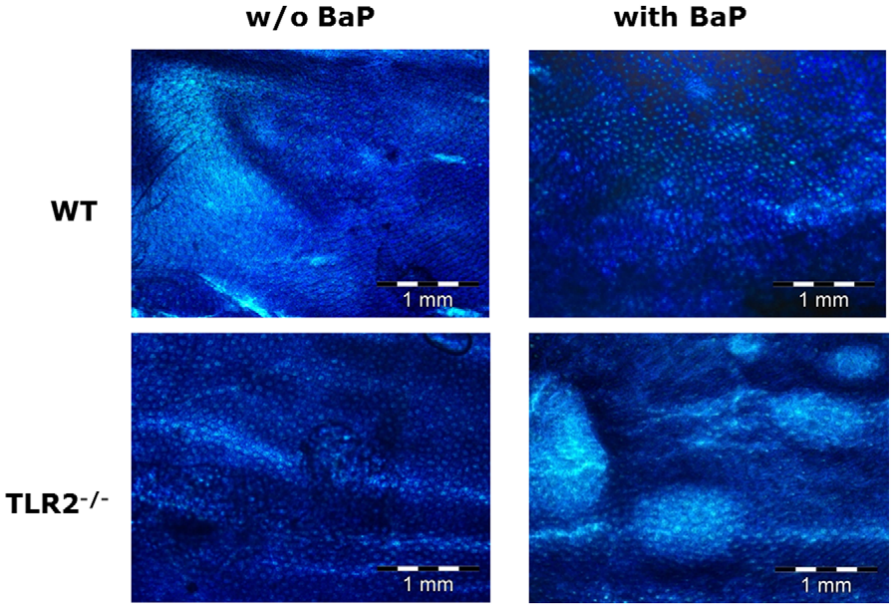
Morphology of colon mucosa from TLR2^−/−^ and wild-type mice with and without BaP feeding. Histological view on methylene-blue stained colon mucosa of mice that were either deficient for functional TLR2 (or not and that were either fed with BaP for 21 days or not. (A) wild-type mice fed corn-oil soaked chow (control diet) (B) wild-type mice fed with BaP (C) TLR2^−/−^ mice on control diet (D) TLR2^−/−^ mice on BaP diet. In the two groups of mice that were fed with BaP, only the colon mucosa of TLR2^−/−^ showed a dense surface coverage with polyps (D), indicating hyperproliferation of the colon mucosa tissue. Bar = 1 mm.

Only the surface of colon mucosa of TLR2^−/−^ animals that were fed with BaP displayed polyp-like structures, but not the colons of the animals from the other three groups (Fig. 4). No preneoplastic foci were seen in any of the colonic tissues.

## Discussion

In face of the carcinogenic potential of PAH, of which BaP is the best investigated one [9], considerable efforts were undertaken to understand the mechanisms by which PAH causes cancer development in different organs. Since the ground-breaking study by Wattenberg and colleagues [20], numerous studies have been carried out to clarify the role of hemethiolate monooxygenases (CYPs) in detoxification of PAH in the intestine. These studies have been related to identification and allocation of different PAH-metabolizing CYPs [21], to transcriptional and translational control of their expression [22], [23] and to regulation of their activity by xenobiotics from industrial processes [8] or dietary intake [24]–[26]. Today, it is clear that CYP1A1 is the key enzyme in metabolizing PAH (together with CYP1B1 and some CYP3A enzymes) [15], [27], [28], while CYP1A2 predominantly converts polar carcinogenic amines [29]. The intestinal expression of murine CYP1A1 is considered to be minimal under non-stimulated conditions and might depend on flavonoid content in the chow [21]. As observed previously [16], CYP1A2 expression was constitutively higher in the liver compared to that in the intestine, but remained unchanged after BaP feeding, though a compensatory CYP1A2 induction in *cyp1a1^−/−^* mice was reported previously [17]. Already under non-stimulated conditions, a significant suppression of CYP1A1 mRNA expression was measured in TLR2^−/−^ mice in the current study (Table 1). In contrast to that, this difference was not noted at the protein level, which might be explained by an increased protein stability phenomenon. The results from the intervention part of the study, in which both types of genotypes (TLR2^−/−^ and WT) were fed BaP, which is a strong inducer of CYP1, confirmed our working hypothesis that the AHR-ARNT system regulating intestinal CYP1A expression [12] depends virtually on intact TLR2 signaling.

In line with previous findings [21], the initially low expression of both mRNA and protein of CYP1A1 in the murine intestine can considerably be increased by BaP feeding in animals with a functional TLR2 receptor. This induction of hydroxylase activity results in induction of both metabolic activation and detoxication of PAH. Metabolic activation, generating reactive intermediates (epoxides) capable of binding to DNA, was considered for decades to be the key pro-carcinogenic process [30]. This concept seemed substantiated by studies on mice with high levels of active CYP1A1 (due to a high affinity AHR), which were more susceptible to cancers and genotoxicity when the PAH was brought in direct contact with the corresponding organs, but not after oral, systemic application [31], [32]. However, BaP feeding (125 mg/kg/day) results in much higher acute toxicity, lethality, and formation of BaP-DNA adducts in the gastrointestinal tract, liver, and other organs of CYP1A1^−/−^ mice [17]. Hence, in the intact animal, intestinal CYP1A1 activity seems important in elimination and detoxification of PAH (and other toxic substrates of CYP1A1) and is, in all likelihood, an important factor in cancer prevention, especially when the activity of phase II enzymes is taken into consideration [11], [17]. The assumption that a high activity of CYPs in the gastrointestinal tract is protective in cancer development is also substantiated by a study in which significantly suppressed levels of three CYPs were found in non-malignant intestinal tissue of patients with adenoma compared to healthy controls [33].

Based on the findings of the current study, the protection of inner organs from the effects of BaP feeding by intestinal CYP1A1 [34] depends – by a so far unidentified mechanism – on the functional activation of the TLR2 receptor and, therefore, on the presence of an intestinal microflora providing ligands for TLR2. This concept is substantiated by the current study as CYP1A1 is induced in the liver of TLR2^−/−^ mice, which lack this intestinal “shield”. Due to the absence of intestinal detoxication in these animals, a higher concentration of BaP might reach the liver and might there induce CYP1A1 expression by the known AHR/ARNT pathway. However, this induction seems not to be sufficient as the TLR2^−/−^ mice – in contrast to the WT mice - were incapable of clearing BaP from their circulation 16 h after cessation of BaP feeding. Hence, the hepatic induction of CYP1A1 by the permeating BaP seems not to be able to compensate for the absence of intestinal CYP1A1 activity in TLR2^−/−^ animals.

Previous studies indicate a protective role of TLR2 in the development of intestinal cancers. TLR2 signaling is a protective factor against colitis-induced cancer [35] and the absence of bacteria from the intestinal lumen results in polyp formation, as seen in studies with gnotobiotic mice [36]. The formation of these pre-carcinogenic proliferations, as also observed in the current study, might be based on a lack of intestinal CYP1A1 activity due to absent TLR2 ligands, which are in first line cell wall components of both Gram-positive and Gram-negative commensal bacteria harbored in the intestinal lumen [37]. The knowledge on the effect of TLR2 mutations on the development of malignant diseases in humans is limited so far. In a Japanese population, subjects with the −196 to −174del/del genotype of TLR2 were shown to be about six times as susceptible to gastric cancer compared to those with the corresponding WT alleles [38].

The mechanism by which TLR2 affects the AHR-associated regulation of CYP1A expression remains to be clarified. To the best of our knowledge, none of the intracellular transcriptional regulation factors linked to TLR2-activated signaling pathways [6] has been related to regulation of CYP1A1, though it has become clear that both production of interleukin 22 by γδ T cells and their expansion depend on AHR availability [39] and mice that lack AHR are much more susceptible to infections with *Toxoplasma gondii* [40], suggesting a relevant role for AHR/ARNT signaling in immune response.

In the current study, the observed dependency of CYP1A1 expression on TLR2 functionality is very unlikely to be due to a malfunction of the AHR/ARNT signaling pathway as an artifact of TLR2 deletion for several reasons: (i) both the TLR2^−/−^ mice and the WT animals carry *Ahr* alleles that result in intestinal mucosa proteins of identical molecular mass (Figure 2). Therefore, the prevalence of different *Ahr* alleles, which might result in the formation of proteins with different ligand affinities [41] can be excluded. (ii) Both the mRNA expression levels of both genes and the corresponding intestinal protein concentrations were unchanged in TLR2^−/−^ animals compared to WT mice (Table 2, Figure 2) (iii) CYP1A2, which also depends on AHR/ARNT regulation [10], is expressed at high levels in the liver and also moderately in the intestine. This would not be the case if the AHR functionality would be impaired; (iv) CYP1A1 is induced in the liver of TLR2^−/−^ mice after exposure to BaP, indicating a well-functioning regulation by AHR/ARNT in this organ; (v) none of the genes that were shown to be altered by genetic deletion of AHR in mice [42] were found to be significantly changed in the TLR2^−/−^ mice (Table 1). This composite evidence strongly points towards a principal availability of an intact AHR/ARNT signaling pathway in the used TLR2^−/−^ mice model.

Besides detoxication of polyaromatic xenobiotics, CYP1A1 is capable of converting endogenous arachidonic acid (AA) into a spectrum of different epoxyeicosatrienoic acids (EETs) and hydroxyeicosatetraenoic acids (HETEs) (i.e. 19-HETE), in a number of organs, including the intestine [43]–[45]. The production of immunomodulating HETEs and EETs [46], [47] by CYP1A1 might explain its link to TLR2 as a sensor for intestinal bacteria. An active role of CYP1-generated AA metabolites in TLR2 deficiency is emphasized by the fact that phospholipase A2, the activity of which results in promotion of AA liberation, is significantly induced in TLR2^−/−^ mice (Table 1). This induction might be explained by a lack of a negative feed-back signal due to the suspected incapability of the animals to form CYP1A1-associated AA metabolites, as observed for other substrates of this enzyme [12].

In conclusion, CYP1A1-facilitated carcinogen detoxication might be under control of TLR2-dependent signaling. Whether the activity of intestinal CYP1A can be influenced by TLR2 ligands and by which molecular mechanisms TLR2 regulates CYP1A expression in the intestine needs to be clarified in future studies. Experiments with gene knockout animals, which show the involvement of other TLRs and intracellular molecules that are related to TLR signaling, might contribute to the understanding of the interaction between TLR2 and CYP1A1 in the intestine.

## References

[1] Jin MS, Lee JO (2008). Structures of the toll-like receptor family and its ligand complexes.. Immunity.

[2] Barton GM, Kagan JC (2009). A cell biological view of Toll-like receptor function: regulation through compartmentalization.. Nat Rev Immunol.

[3] Uematsu S, Akira S (2008). Toll-like receptors (TLRs) and their ligands.. Handb Exp Pharmacol.

[4] Kawai T, Akira S (2006). Innate immune recognition of viral infection.. Nat Immunol.

[5] O'Neill LA, Bowie AG (2007). The family of five: TIR-domain-containing adaptors in Toll-like receptor signalling.. Nat Rev Immunol.

[6] Mele T, Madrenas J (2010). TLR2 signalling: At the crossroads of commensalism, invasive infections and toxic shock syndrome by *Staphylococcus aureus*.. Int J Biochem Cell Biol.

[7] Rafter J (2004). The effects of probiotics on colon cancer development.. Nutr Res Rev.

[8] Waldman JM, Lioy PJ, Greenberg A, Butler JP (1991). Analysis of human exposure to benzo(a)pyrene via inhalation and food ingestion in the Total Human Environmental Exposure Study (THEES).. J Expo Anal Environ Epidemiol.

[9] Alexandrov K, Rojas M, Satarug S (2010). The critical DNA damage by benzo(a)pyrene in lung tissues of smokers and approaches to preventing its formation.. Toxicol Lett.

[10] Gu J, Horikawa Y, Chen M, Dinney CP, Wu X (2008). Benzo(a)pyrene diol epoxide-induced chromosome 9p21 aberrations are associated with increased risk of bladder cancer.. Cancer Epidemiol Biomarkers Prev.

[11] Nebert DW, Dalton TP, Okey AB, Gonzalez FJ (2004). Role of aryl hydrocarbon receptor-mediated induction of the CYP1 enzymes in environmental toxicity and cancer.. J Biol Chem.

[12] Chiaro CR, Patel RD, Marcus CB, Perdew GH (2007). Evidence for an aryl hydrocarbon receptor-mediated cytochrome p450 autoregulatory pathway.. Mol Pharmacol.

[13] Nebert DW, Roe AL, Dieter MZ, Solis WA, Yang Y (2000). Role of the aromatic hydrocarbon receptor and [Ah] gene battery in the oxidative stress response, cell cycle control, and apoptosis.. Biochem Pharmacol.

[14] Wooten RM, Ma Y, Yoder RA, Brown JP, Weis JH (2002). Toll-like receptor 2 is required for innate, but not acquired, host defense to Borrelia burgdorferi.. J Immunol.

[15] Uno S, Dalton TP, Dragin N, Curran CP, Derkenne S (2006). Oral benzo[a]pyrene in Cyp1 knockout mouse lines: CYP1A1 important in detoxication, CYP1B1 metabolism required for immune damage independent of total-body burden and clearance rate.. Mol Pharmacol.

[16] Hruz T, Laule O, Szabo G, Wessendorp F, Bleuler S (2008). Genevestigator v3: a reference expression database for the meta-analysis of transcriptomes.. Adv Bioinformatics.

[17] Uno S, Dalton TP, Derkenne S, Curran CP, Miller ML (2004). Oral exposure to benzo[a]pyrene in the mouse: detoxication by inducible cytochrome P450 is more important than metabolic activation.. Mol Pharmacol.

[18] Wagnerberger S, Schäfer C, Bode C, Parlesak A (2006). Saturation of retinol-binding protein correlates closely to the severity of alcohol-induced liver disease.. Alcohol.

[19] Smyth GK, Gentleman R, Carey V, Dudoit S, Irizarry R, Huber W (2005). Limma: linear models for microarray data.. Bioinformatics and Computational Biology Solutions using R and Bioconductor.

[20] Wattenberg LW, Leong JL, Strand PJ (1962). Benzpyrene hydroxylase activity in the gastrointestinal tract.. Cancer Res.

[21] Uno S, Dragin N, Miller ML, Dalton TP, Gonzalez FJ (2008). Basal and inducible CYP1 mRNA quantitation and protein localization throughout the mouse gastrointestinal tract.. Free Radic Biol Med.

[22] Traber PG, McDonnell WM, Wang W, Florence R (1992). Expression and regulation of cytochrome P-450 I genes (CYP1A1 and CYP1A2) in the rat alimentary tract.. Biochim Biophys Acta.

[23] Zhang QY, Wikoff J, Dunbar D, Fasco M, Kaminsky L (1997). Regulation of cytochrome P4501A1 expression in rat small intestine.. Drug Metab Dispos.

[24] Lindeskog P, Overvik E, Nilsson L, Nord CE, Gustafsson JA (1988). Influence of fried meat and fiber on cytochrome P-450 mediated activity and excretion of mutagens in rats.. Mutat Res.

[25] Fontana RJ, Lown KS, Paine MF, Fortlage L, Santella RM (1999). Effects of a chargrilled meat diet on expression of CYP3A, CYP1A, and P-glycoprotein levels in healthy volunteers.. Gastroenterology.

[26] Ito S, Chen C, Satoh J, Yim S, Gonzalez FJ (2007). Dietary phytochemicals regulate whole-body CYP1A1 expression through an aryl hydrocarbonreceptor nuclear translocator-dependent system in gut.. J Clin Invest.

[27] Guengerich FP (2000). Metabolism of chemical carcinogens.. Carcinogenesis.

[28] Burk O, Koch I, Raucy J, Hustert E, Eichelbaum M (2004). The induction of cytochrome P450 3A5 (CYP3A5) in the human liver and intestine is mediated by the xenobiotic sensors pregnane X receptor (PXR) and constitutively activated receptor (CAR).. J Biol Chem.

[29] Butler MA, Iwasaki M, Guengerich FP, Kadlubar FF (1989). Human P450PA (P450-1A2), the phenacetin O-deethylase, is primarily responsible for the hepatic 3-demethylation of caffeine and N-oxidation of carcinogenic amines.. Proc Natl Acad Sci USA.

[30] Grover PL, Sims P (1968). Enzyme-catalysed reactions of polycyclic hydrocarbons with deoxyribonucleic acid and protein in vitro.. Biochem J.

[31] Nebert DW (1989). The Ah locus: genetic differences in toxicity, cancer, mutation, and birth defects.. Crit Rev Toxicol.

[32] Conney AH (2003). Enzyme induction and dietary chemicals as approaches to cancer chemoprevention: the Seventh DeWitt S. Goodman Lecture.. Cancer Res.

[33] Bergheim I, Bode C, Parlesak A (2005). Decreased expression of cytochrome P450 protein in non-malignant colonic tissue of patients with colonic adenoma.. BMC Gastroenterol.

[34] Shi Z, Dragin N, Gálvez-Peralta M, Jorge-Nebert LF, Miller ML (2010). Organ-specific roles of CYP1A1 during detoxication of dietary benzo[a]pyrene.. Mol Pharmacol.

[35] Lowe EL, Crother TR, Rabizadeh S, Hu B, Wang H (2010). Toll-like receptor 2 signaling protects mice from tumor development in a mouse model of colitis-induced cancer.. PLoS One.

[36] Ozaki A, Morishita Y, Oowada T, Itagaki S, Mizutani T (1998). Inhibition of polyposis in the small intestine of BALB/c mice by intestinal bacteria.. Cancer Lett.

[37] Zähringer U, Lindner B, Inamura S, Heine H, Alexander C (2008). TLR2 - promiscuous or specific? A critical re-evaluation of a receptor expressing apparent broad specificity.. Immunobiology.

[38] Tahara T, Arisawa T, Wang F, Shibata T, Nakamura M (2007). Toll-like receptor 2 −196 to 174del polymorphism influences the susceptibility of Japanese people to gastric cancer.. Cancer Sci.

[39] Martin B, Hirota K, Cua DJ, Stockinger B, Veldhoen M (2009). Interleukin-17-producing gammadelta T cells selectively expand in response to pathogen products and environmental signals.. Immunity.

[40] Sanchez Y, Rosado Jde D, Vega L, Elizondo G, Estrada-Muñiz E (2010). The unexpected role for the aryl hydrocarbon receptor on susceptibility to experimental toxoplasmosis.. J Biomed Biotechnol.

[41] Poland A, Palen D, Glover E (1994). Analysis of the four alleles of the murine aryl hydrocarbon receptor.. Mol Pharmacol.

[42] Tijet N, Boutros PC, Moffat ID, Okey AB, Tuomisto J (2006). Aryl hydrocarbon receptor regulates distinct dioxin-dependent and dioxin-independent gene batteries.. Mol Pharmacol.

[43] Nebert DW, Karp CL (2008). Endogenous functions of the aryl hydrocarbon receptor (AHR): intersection of cytochrome P450 1 (CYP1)-metabolized eicosanoids and AHR biology.. J Biol Chem.

[44] Zeldin DC, Foley J, Goldsworthy SM, Cook ME, Boyle JE (1997). CYP2J subfamily cytochrome P450s in the gastrointestinal tract: expression, localization, and potential functional significance.. Mol Pharmacol.

[45] Schwarz D, Kisselev P, Ericksen SS, Szklarz GD, Chernogolov A (2004). Arachidonic and eicosapentaenoic acid metabolism by human CYP1A1: highly stereoselective formation of 17(R),18(S)-epoxyeicosatetraenoic acid.. Biochem Pharmacol.

[46] Node K, Huo Y, Ruan X, Yang B, Spiecker M (1999). Anti-inflammatory properties of cytochrome P450 epoxygenase-derived eicosanoids.. Science.

[47] Miyata N, Roman RJ (2005). Role of 20-hydroxyeicosatetraenoic acid (20-HETE) in vascular system.. J Smooth Musc Res.

